# A prospective marker for the prediction of postoperative deep venous thrombosis: Neutrophil extracellular traps

**DOI:** 10.3389/fcell.2022.1071550

**Published:** 2022-11-16

**Authors:** Yin Li, Qinyi Jiang, Xiaohua Zhou, Mengyuan Wu, Jian Chen, Hao Liu, Siming Dai, Ziyang Zheng, Xuan Zhao, Chenxi Zhang, Zhuoying Shi, Haitao Zhang, Jinyu Gu, Zhenfei Huang, Guoyong Yin, Shujie Zhao

**Affiliations:** ^1^ Department of Orthopaedics, The First Affiliated Hospital of Nanjing Medical University, Nanjing, Jiangsu, China; ^2^ Jiangsu Institute of Functional Reconstruction and Rehabilitation, Nanjing, Jiangsu, China; ^3^ Spinal Cord Disease Research Center, Nanjing Medical University, Nanjing, Jiangsu, China; ^4^ Department of Orthopaedics, People’s Hospital Affiliated to Jiangsu University, Zhenjiang, Jiangsu, China; ^5^ Department of Anesthesia and Perioperative Medicine, The First Affiliated Hospital of Nanjing Medical University, Nanjing, Jiangsu, China

**Keywords:** deep venous thrombosis, neutrophil extracellular traps, lumbar fracture, predicting marker, citrullinated histone H3

## Abstract

Deep venous thrombosis (DVT) is a common medical complication in patients with lumbar fractures. The current study aimed to investigate the predictive value of neutrophil extracellular traps (NETs) in postoperative DVT formation in patients with lumbar fractures and to develop a nomogram relating clinical admission information for prediction. Patients who underwent open reduction and pedicle screw internal fixation in the treatment of single-segment lumbar fracture in the Department of Spine Surgery, the First Affiliated Hospital of Nanjing Medical University, from December 2020 to June 2022 were enrolled in this study. Baseline data and laboratory results were collected from enrollees, and the primary study endpoint event was the occurrence of DVT in patients after surgery. Multivariable logistic regression analysis was used to identify risk factors associated with higher odds of DVT after surgery. A nomogram was constructed using the results of the multivariable model. The calibration plot and receiver operating characteristics (ROC) curve were used to show the satisfactory predictive capacity of the model. Of these 393 patients who did not have DVT preoperatively, 79 patients developed it postoperatively, and 314 did not, respectively. Multivariate analysis showed that higher body mass index (BMI) (BMI between 24 and 28: RR = 1.661, 95% CI = 0.891–3.094; BMI ≤28: RR = 5.625, 95% CI = 2.590–12.217; reference: BMI <24), neutrophils (RR = 1.157, 95% CI 1.042–1.285), D-dimer (RR = 1.098, 95% CI 1.000–1.206), and citrullinated histone H3 (CitH3) (RR = 1.043, 95% CI 1.026–1.060) were independent risk factors for postoperative DVT. Using the multivariable analysis, we then constructed a nomogram to predict DVT, which was found to have an area under the curve of 0.757 (95% CI = 0.693–0.820). Calibration plots also showed the satisfied discrimination and calibration of the nomogram. In conclusion, patients with lumbar fractures with postoperative DVT had higher levels of NETs in the circulation preoperatively compared to those without postoperative DVT. Furthermore, based on BMI, D-dimer, neutrophils, and CitH3, we developed a predictive model to predict postoperative DVT incidence in these patients.

## Introduction

Besides tremendously impacting the patient’s quality of life, lumbar fractures burden families and society. Spine fractures account for 5%–6% of total body fractures, with the lumbar spine being a high prevalence location for spine fractures (Liu et al., 2012; Wang et al., 2012). Deep venous thrombosis (DVT) is one of the most common complications of lumbar fractures. Studies have shown that the incidence of venous thromboembolism after spine surgery ranges from 0.3% to 31% (Fineberg et al., 2013; Mosenthal et al., 2018). If not timely diagnosed or treated, DVT would develop into post-thrombotic syndrome, which leads to ulcers, necrosis, and pigmentation in lower limbs (Galanaud et al., 2018). Furthermore, due to the sizeable vascular diameter of the proximal lower extremity, the thrombus in this site is prone to dislodge and develop into fatal pulmonary thromboembolism (PE) (Qiu et al., 2021). Therefore, to reduce the incidence and mortality of DVT and ease the socioeconomic stress, there is an urgent demand for discovering predictors of DVT and taking corresponding precautions in the perioperative period of spine surgery besides improving the diagnostic technique (Le Gal et al., 2017; Ruskin, 2018; Wang et al., 2021).

Neutrophil extracellular traps (NETs), released by activated neutrophils, contain double-stranded DNA, histone, and granule proteins, which include neutrophil elastase (NE), cathepsin G, and myeloperoxidase (MPO) (Urban et al., 2009; Papayannopoulos, 2018; Yang and Liu, 2021). Increased NETs can be a double-edged sword. On the one hand, these components are essential to innate immune defense against pathogens (Jorch and Kubes, 2017). On the other hand, when not well-regulated, NETs have the potential to facilitate inflammatory pathologies of several diseases, such as deep vein thrombosis, by providing a scaffold for blood cells to attach to and activating coagulation pathways (Martinod et al., 2013; Savchenko et al., 2014). Researchers have recently conducted preliminary explorations of the relationship between NETs and thrombosis (Martinod et al., 2013; Kimball et al., 2016; Liu et al., 2021). It is assumed that the plasma level of citrullinated histone H3 (CitH3), a specific marker of NETs, could assist in diagnosing and predicting DVT in traumatic lower extremity fractures (Liu et al., 2021). Nevertheless, to our knowledge, the relationship between the preoperative level of NETs and postoperative DVT in patients undergoing spine surgery for lumbar fractures has not been fully elucidated.

In the present study, NETs released by neutrophils were observed in the circulation of patients with lumbar fractures. Our results also demonstrated that high levels of NETs in patients with lumbar fractures before surgery predict an increased risk of postoperative DVT. Notably, we developed a model for predicting the formation of DVT after surgery for lumbar fractures, and this model might be put into clinical application.

## Materials and methods

### Patient recruitment and study design

**FIGURE 1:**
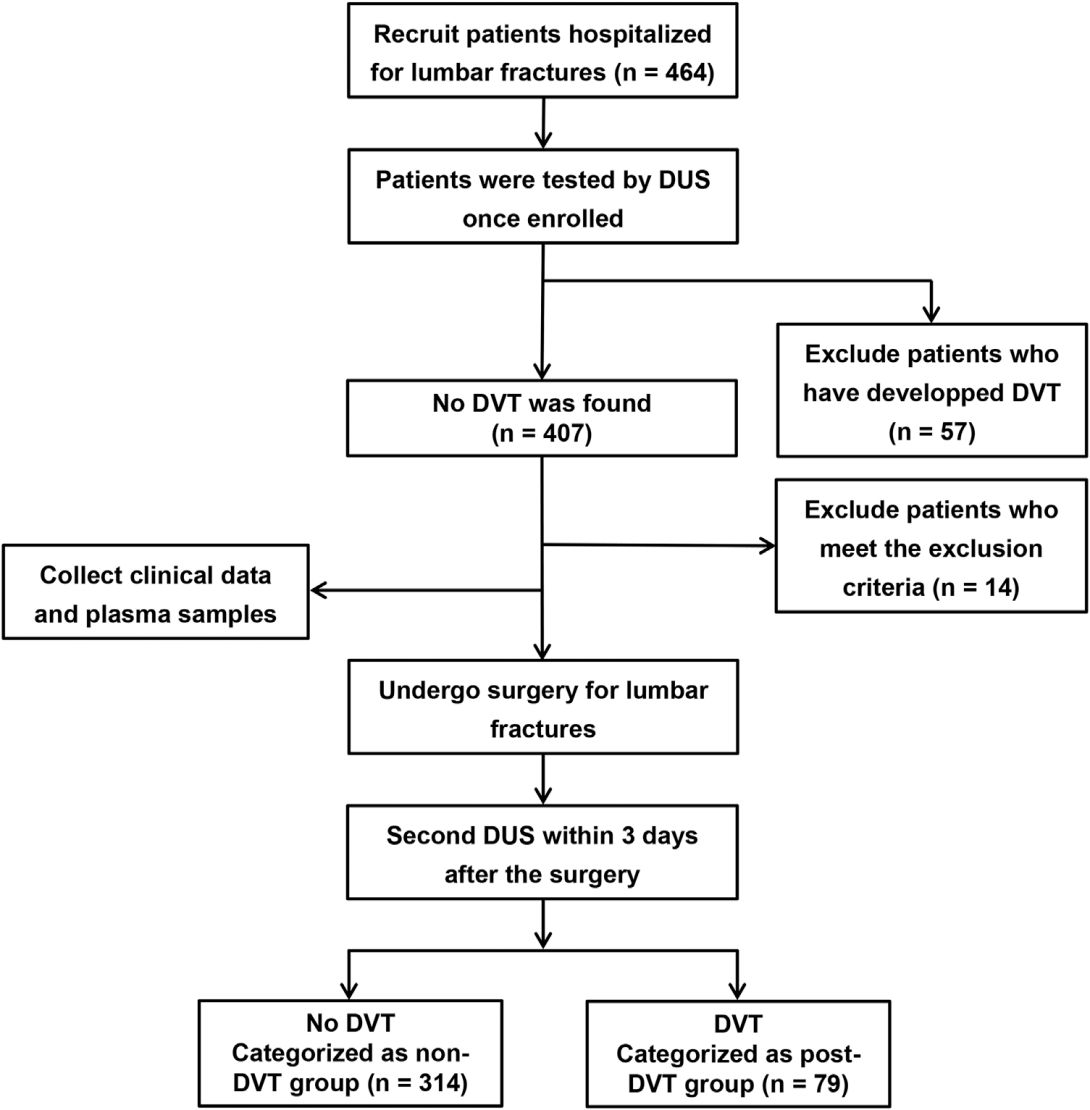
Flow chart of classification of non-DVT and post-DVT groups.

Our study included 393 patients who underwent open reduction and pedicle screw internal fixation in the treatment of single-segment lumbar fracture in the Department of Spine Surgery, the First Affiliated Hospital of Nanjing Medical University, from December 2020 to June 2022. Study participants were excluded if they met the following exclusion criteria: age <18 years; pregnant; combined with thrombosis or spinal cord injury; previous history of another vascular embolism such as myocardial infarction; under treatment with anticoagulation and antiplatelet such as heparin, warfarin, and aspirin within 4 weeks before admission; blood coagulation dysfunction; immune dysfunction; history of varicose veins; cardiovascular disease; infection; tumor and incomplete medical records. Once hospitalized, all participants routinely underwent doppler ultrasound (DUS) scanning of bilateral lower extremities to distinguish DVT or not (Suenaga et al., 2010; Hansrani et al., 2017). The same procedure was performed within 3 days after the operation. 79 Patients who were not found DVT when admitted but developed it after surgery were enrolled in the post-DVT group. The non-DVT group enrolled 314 patients with no thrombosis before and after surgery. Clinical and laboratory data were collected, including age, sex, body mass index (BMI), chronic medical diseases (hypertension, diabetes), hemoglobin, platelets, monocytes, neutrophils, lymphocytes, D-dimer, and CitH3, obtained during hospitalization. Figure 1 shows the flow chart of grouping. The study was approved by the Ethical Committee of The First Affiliated Hospital of Nanjing Medical University.

### Blood sample analysis

Blood was collected from participants on the second day after hospitalization. 4 ml venous whole blood was anticoagulated with an EDTA tube, gently mixed into a 15 ml centrifuge tube, and centrifugated at 3,000 rpm at 4°C for 20 min. Then the plasma was obtained and stored at −80°C for subsequent use. CitH3 was quantified in plasma using the Citrullinated Histone H3 (clone 11D3) ELISA Kit (Cayman, 501,620) according to the manufacturer’s instructions. All samples were repeated in triplicate. In addition, each patient was inspected for blood routine examination on admission. So we obtain the results directly from the laboratory department.

### Neutrophil isolation and neutrophil extracellular traps immunofluorescence

Polymorphonuclear neutrophils (PMNs) were isolated with Human StraightFrom Whole Blood CD15 microbeads kits (130-091-058, Miltenyi Biotech, Germany). Briefly, after erythrocyte lysis, the blood cells were incubated in the presence of CD15 microbeads. Blood cells were washed with separation buffer and centrifuged at room temperature at 300 g for 10 min. The pellets containing magnetically labeled cells were resuspended in a separation buffer and applied to the equilibrated whole blood column placed on the QuadroMACS. After extensive washing, the column was removed from the magnetic separator, and labeled cells were eluted using a whole-blood elution buffer. Finally, the PMNs were centrifuged at 300 g for 10 min and resuspended in Hank’s buffer with calcium and magnesium (Carminita et al., 2021).

After isolation, 1 × 10^5^ PMNs were seeded onto a glass bottom cell culture dish coated with 0.001% poly-l-lysine (Sigma-Aldrich, P4707) and cultured at 37°C and 5% CO_2_ for 1 h. NETs formation by adherent PMNs was induced by stimulation with 20 nM Phorbol 12-myristate 13-acetate (PMA) (Sigma-Aldrich, P1585) for 1 h at 37°C. Then, the cells were fixed with 4% paraformaldehyde for 10 min at room temperature, followed by washing with PBS and blocking with Blocking Buffer (P0235, Beyotime, China). The cells were incubated overnight in the dark at 4°C with an anti-Histone H3 (citrulline R2 + R8 + R17) antibody (Abcam 5,103, diluted 1:1000) and an anti-Human/Mouse MPO Antibody (R&D Systems, AF3667, 10 µg/ml). After washing, the corresponding secondary antibodies conjugated to green and red fluorescent dyes were added to the cells, and nuclei were stained with DAPI. Images used in the figures were captured using a confocal microscope (Zeiss LSM710, Heidenheim, Germany).

### Statistical analysis

SPSS version 26.0 (IBM Corporation, Armonk, NY) was used for statistical data analysis. Continuous variables were displayed as mean ± standard deviation. Categorical variables were displayed as counts (n) and percentages (%). The independent sample *t*-test evaluated differences in continuous variables between groups, and the Chi-square test was applied for categorical variables. Multivariate logistic regression analysis was performed to calculate the relative risk (RR) and the 95% confidence interval (CI). A nomogram was established to predict postoperative DVT. The calibration curve was used to display the predictive value of the prognostic models. Additionally, the receiver operating characteristic (ROC) curve was constructed, and the area under the ROC curve (AUC) was calculated to illustrate the value for predicting DVT. *p* < 0.05 was considered statistically significant.

## Results

### Demographic data of each experimental group

The baseline data of the study subjects are displayed in Table 1. There was no significant difference in age, gender, or chronic medical diseases (hypertension, diabetes) between non-DVT and post-DVT groups. Compared with the non-DVT group, patients in the post-DVT group experienced significantly increased levels of BMI (*p* < 0.001).

**TABLE 1:** Demographic data of patients.

	Non-DVT (*n* = 314)	Post-DVT (*n* = 79)	*p*-Value
Age, years	58.3 ± 12.7	59.4 ± 11.8	0.516
Gender, n (%)			0.208
Male	164 (52.2)	35 (44.3)	
Female	150 (47.8)	44 (55.7)	
BMI, kg/m^2^			<0.001
<24	152	24	
24-28	131	35	
≥28	31	20	
Hypertension, n (%)	90 (28.7)	24 (30.4)	0.764
Diabetes, n (%)	47 (15.0)	10 (12.7)	0.602

*p* < 0.05 was considered statistically significant; BMI, body mass index.

### Patients in the post-deep venous thrombosis group had higher preoperative blood concentrations of citrullinated histone H3

**FIGURE 2:**
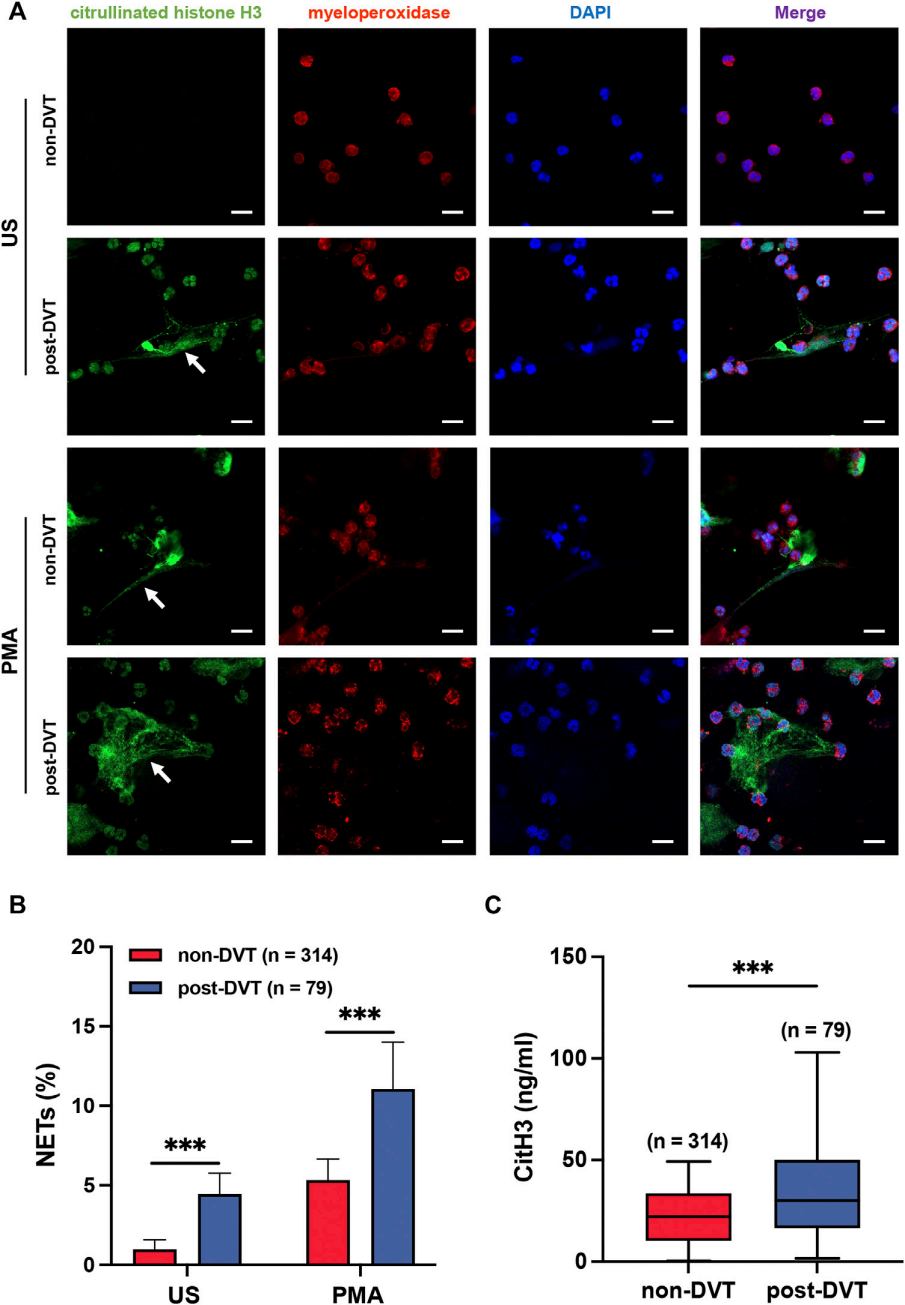
Neutrophils from the blood of patients in the post-DVT group form more NETs compared to the non-DVT group. **(A)** Representative images of Immunofluorescence staining of peripheral blood neutrophils from patients in non-DVT and post-DVT groups for MPO (red) and CitH3 (green) are shown, and the cell nuclei were stained with DAPI (blue). The white arrows indicate the typical NETs structure. Scale bars, 30 μm. US, unstimulated. **(B)** Results of the statistical analysis show the percentage of NETs in each group (*n* = 314 in the non-DVT group and *n* = 79 in the post-DVT group). ***p < 0.001. **(C)** Plasma samples were analyzed for CitH3 (*n* = 314 in the non-DVT group and *n* = 79 in the post-DVT group). ***p < 0.001.

To demonstrate the reticular structure of NETs, we stained MPO red and CitH3 green (Figure 2A). We used the proportion of green area (CitH3) in a field of view to measure the degree of NETs formation (Figure 2B). In the unstimulated (US) group, neutrophils isolated from the blood of patients in the post-DVT group showed a significant increase in the percentage of NETs in comparison with the corresponding control group (4.5 ± 1.0 versus 1.0 ± 0.6, *p* < 0.001). As predicted, this finding was amplified by the stimulation of PMA, and the result of the PMA group was similar to that of the US group (11.1 ± 2.9 versus 5.3 ± 1.3, *p* < 0.001). Moreover, for the plasma CitH3, the concentration in the post-DVT group was 35.18 ± 23.79 ng/ml, which was statistically higher than 22.49 ± 13.89 ng/ml in the non-DVT group (*p* < 0.001) (Figure 2C).

### Plasma citrullinated histone H3 acts as a predictive marker for deep venous thrombosis

**FIGURE 3:**
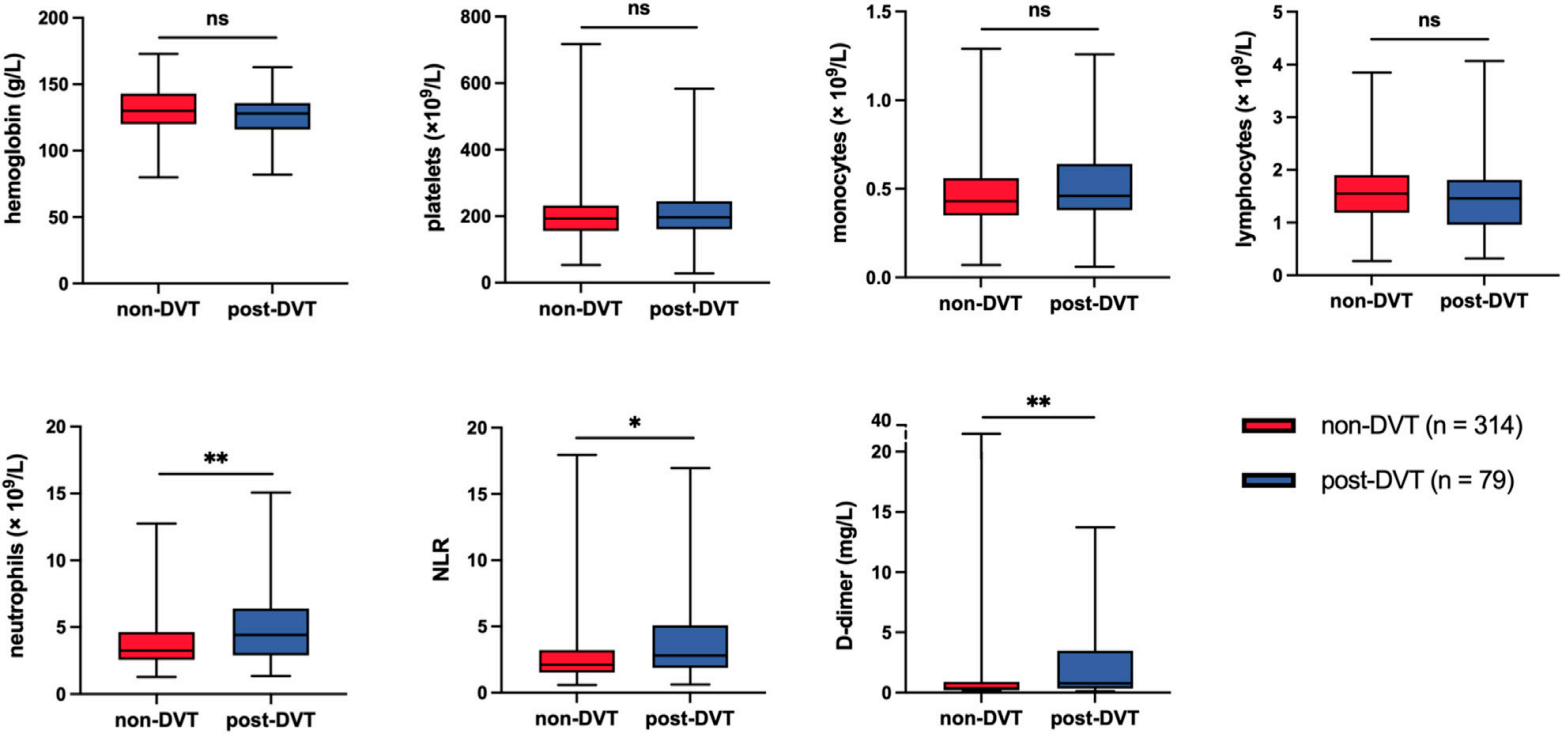
Statistical analysis of laboratory data after admission to hospital between the two study groups. ns, no significance; *p < 0.05; **p < 0.01; NLR, neutrophil–lymphocyte ratio.

All patients were routinely inspected for blood routine examination and D-dimer upon admission, and we obtained the clinical data of these indices from the laboratory department. As shown in Figure 3, compared with the non-DVT group, patients in the post-DVT group experienced significantly increased levels of neutrophils (*p* = 0.001), Neutrophil–lymphocyte ratio (NLR) (*p* = 0.010) and D-dimer (*p* = 0.002). The levels of other parameters, including hemoglobin, platelets, monocytes, and lymphocytes, were not statistically significant between the two groups (Supplementary Table S1).

**FIGURE 4:**
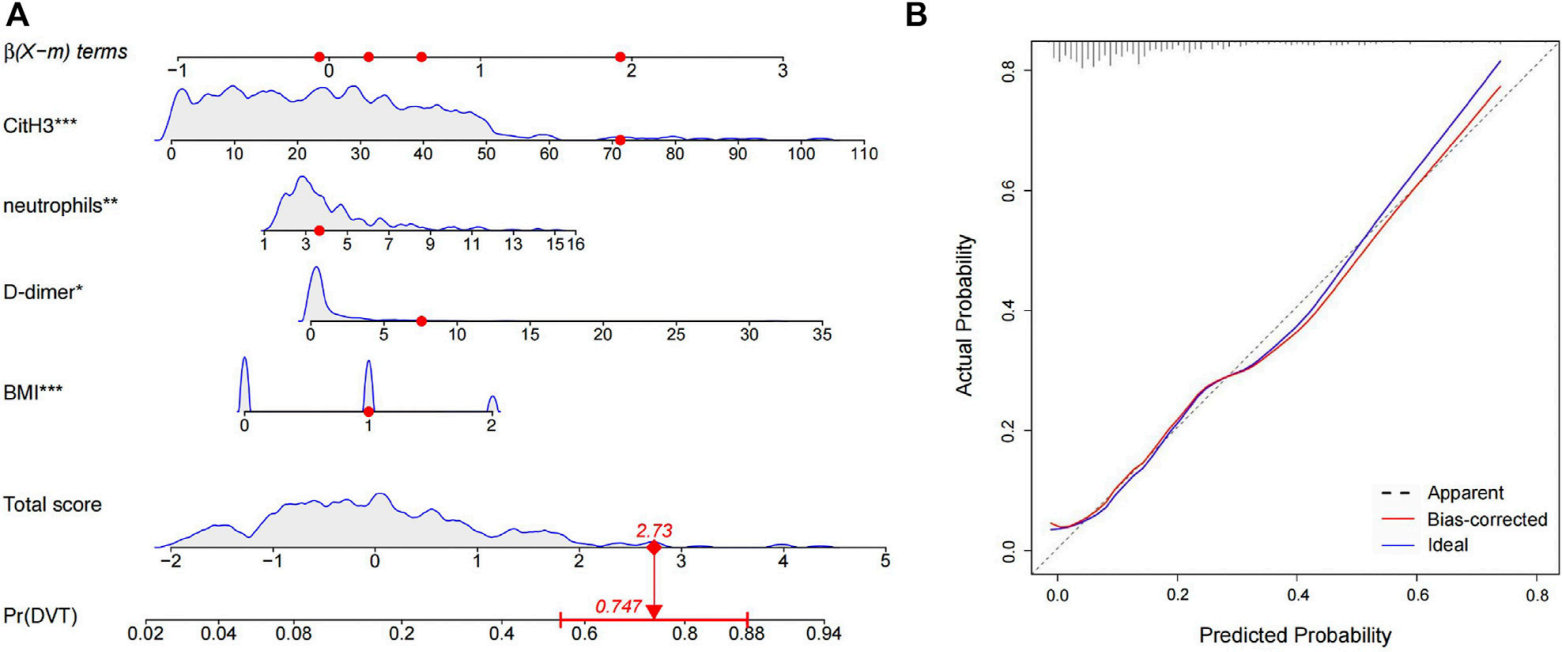
Nomogram and the corresponding calibration curve for predicting the risk of operative DVT. **(A)** Four predictors were involved in the predicting model, and each of them was assigned a graphic score. The sum of these four scores generated a result on the “total score” axis, and the “Pr (DVT)” axis shows the probability of DVT. Red dots represented the scores of a random patient. **(B)** The calibration curve of the predictive model for evaluating the risk of postoperative DVT.

In total, we selected five factors (BMI, neutrophils, NLR, D-dimer, and CitH3) that were significantly different between the two groups to perform multivariate logistic regression analysis. Four predictors remained significant in the multivariate logistic regression model after multivariable adjustment. Table 2 shows the independent predictors of DVT after lumbar surgery, which include BMI, neutrophils, D-dimer, and CitH3. Respectively, BMI between 24 and 28 group (versus BMI <24 group, RR = 1.661, 95% CI = 0.891–3.094), BMI >28 group (versus BMI <24 group, RR = 5.625, 95% CI = 2.590–12.217), D-dimer (RR = 1.098, 95% CI 1.000–1.206), neutrophils (RR = 1.157, 95% CI 1.042–1.285), and CitH3 (RR = 1.043, 95% CI 1.026–1.060) predicted an increased risk of DVT. The Hosmer-Lemeshow test showed the good fitness (X^2^ = 4.765, *p* = 0.782, Nagelkerke *R* ^2^ = 0.236). To visualize the logistic regression model, we constructed a nomogram in which a graphic score was statistically assigned to each significant predictor for quick reference (Figure 4A). The total points of summed scores referred to an individual probability of DVT after lumbar surgery. For instance, red points in Figure 4 showed a patient’s scores for each item, and his probability of deep vein thrombosis after surgery was 74.7%. The calibration plot revealed good predictive accuracy between the nomogram prediction and actual observation (Figure 4B).

**TABLE 2:** Multivariate logistic regression analyses of risk factors for predicting postoperative DVT.

Variables	Standard error	Wald X^2^	RR	95% CI	*p*-Value
BMI					
<24	References				
24–28	0.318	2.552	1.661	0.891–3.094	0.110
≥28	0.396	19.052	5.625	2.590–12.217	<0.001
D-dimer	0.05	3.8	1.098	1.000–1.206	0.051
neutrophils	0.05	7.43	1.157	1.042–1.285	0.006
CitH3	0.01	26.64	1.043	1.026–1.060	<0.001

DVT, deep venous thrombosis; RR, relative risk; CI, confidence interval; BMI, body mass index; CitH3, citrullinated histone H3.

**FIGURE 5:**
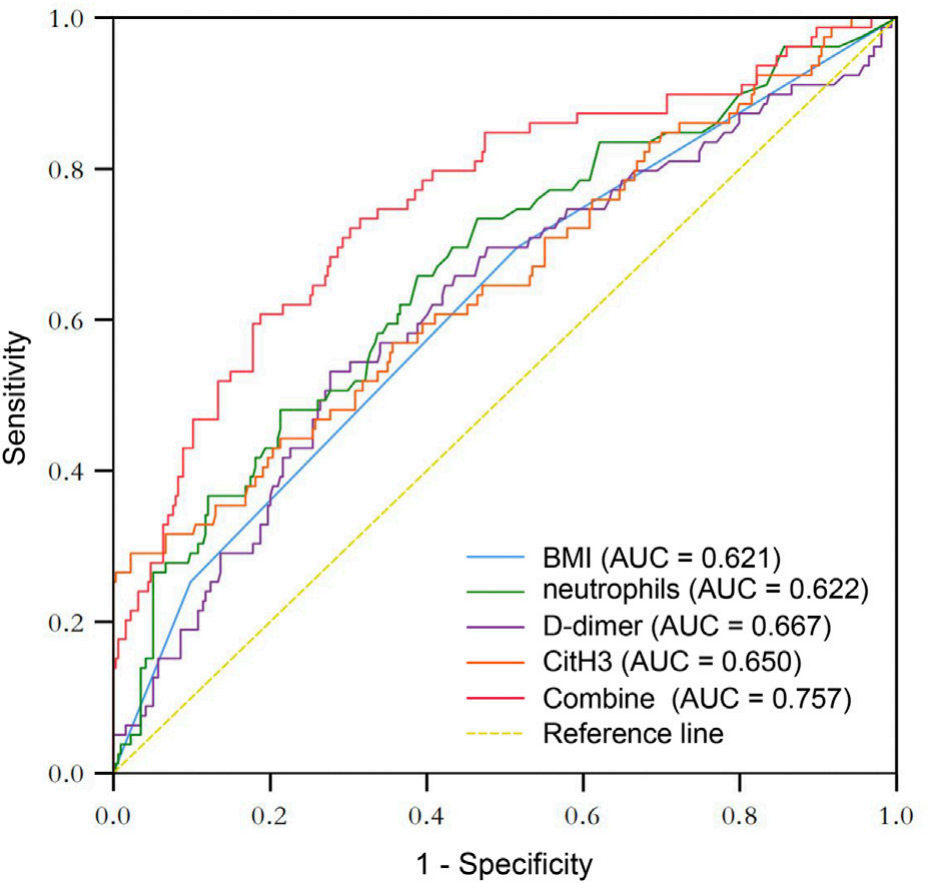
ROC curves of the risk factors in DVT, as well as the combination of these factors in predicting DVT.

As presented in Figure 5, the ROC curve marked in orange demonstrated that the AUC of the plasma level of CitH3 before surgery for predicting DVT was 0.650 (95% CI = 0.577–0.723). The optimal cutoff value for CitH3 was 47.925 ng/ml, and the sensitivity and specificity for predicting DVT were 29.1% and 97.8%, respectively. The ROC curve of CitH3 combined with the other three predictors was represented by a red line with an AUC of 0.757 (95% CI = 0.693–0.820). The sensitivity was 60.8%, and the specificity was 81.2%, respectively. The AUC for the other predictors were as follows: BMI, 0.621 (0.550–0.692); neutrophils, 0.622 (0.550–0.694); D-dimer, 0.667 (0.598–0.736).

## Discussion

DVT, as a perioperative complication with a high incidence in patients with lumbar fractures, seriously affects the prognosis of patients (Chakravarthy et al., 2019). Clinical diagnosis alone is often unreliable and a large percentage of DVT occurring in the hospital are asymptomatic, leading to DVT being easily undiagnosed (Hansrani et al., 2017). Occasionally, the embolus will endanger the patient’s life once it dislodges from the vessel wall causing PE. Hence, finding a marker that can predict DVT in the early stage of fracture is crucial. In this study, we detected the level of NETs in patients with lumbar fractures. We found that a high level of preoperative NETs could predict postoperative DVT, which led us to develop a predictive model consisting of multiple markers, including NETs.

NETs can promote venous thromboembolism through different mechanisms. By providing a scaffold for erythrocytes, leukocytes, platelets, and activated coagulation factors, NETs stimulate the adhesion and aggregation of these substances directly (Lendvai et al., 2013; Martinod et al., 2013; Li et al., 2020). In addition, the polyanionic surface of NETs activates the intrinsic coagulation pathways, and NETs can also bind and induce the expression of tissue factor to initiate the extrinsic coagulation pathways (Iba et al., 2014; Carminita et al., 2021). Meanwhile, NE, the critical protein of NETs, inactivates anticoagulants *in vivo* by decomposition of tissue factor pathway inhibitors and thrombomodulin, thereby exacerbating the coagulation response (Iba et al., 2014; Waisberg et al., 2014).

According to the previous reports in the literature, NETs and DVT are inextricably linked. von Bruhl et al. (2012) found that activated neutrophils and NETs took part in thrombosis after inferior vena cava ligation in mice. Similar results suggest that NETs released by neutrophils are present in thrombi in experimental mice and coronary arteries of patients (Mangold et al., 2015). Circulating levels of nucleosomes and activated neutrophils were notably elevated in patients with DVT, and levels of either were associated with an approximately 3-fold increased risk of developing DVT (van Montfoort et al., 2013; Riegger et al., 2016). Clinical studies have further demonstrated that the circulating level of NETs is significantly increased during acute thrombotic episodes (Diaz et al., 2013; van Montfoort et al., 2013; Lee et al., 2018).

In our predictive model, in addition to NETs, BMI, Neutrophils, and D-dimer were also predictors of postoperative DVT. Patients with higher BMI had an increased risk of DVT after spine surgery, and this study noted that the probability of venous thrombosis increased by 9.4% for every 1 kg/m^2^ increase in BMI (Xu et al., 2020). Patients with high BMI may have relatively more adipose tissue, leading to increased release of inflammatory cytokines and enhanced synthesis of tissue factor, which activates exogenous coagulation pathways and thus promotes thrombus formation (Hunt, 2017). Additionally, obese patients are often associated with hyperlipidemia and slower blood flow than average, further inducing DVT (Vaya et al., 2002). There is a significant relationship between D-dimer and alterations in the hypercoagulability and fibrinolytic system of the organism (Goldenberg et al., 2004; Terpos et al., 2020). With high sensitivity, D-dimer is a well-established diagnostic marker for DVT. Furthermore, a multicenter study showed that the high level of D-dimer is both a diagnostic indicator and a predictor of DVT after spinal surgery (Cohen et al., 2014). Our results further corroborate the effectiveness of D-dimer in predicting DVT. Previous studies have shown that neutrophils act as an indicator of inflammation and contribute to DVT by releasing NETs and neutrophil histone modifications (Brill et al., 2012; Brinkmann, 2018). Notably, there is controversy regarding the relationship between neutrophil counts and venous thrombosis (Martos et al., 2020; Ferrer-Marin et al., 2021; Langiu et al., 2022). Studies suggested that NETs rather than increased neutrophil counts are crucial in promoting DVT formation (Noubouossie et al., 2019). The role of increased neutrophil counts in DVT formation after surgery is still undefined.

Several limitations of this study should be discussed. First, the NETs data in this study were at levels before surgical stimulation and did not examine the expression levels of NETs longitudinally during subsequent thrombolysis in patients. Follow-up studies will be meaningful to detect NETs in the period after surgery. Second, our study has not explored the specific molecular mechanisms of NETs formation. And finally, the conclusions obtained from this study need to be validated by a large sample and multicenter study.

To summarize, this study proposed associations between NETs and postoperative DVT in patients with lumbar fractures, suggesting that intervention with NETs is a promising strategy to prevent DVT. And as stated in the results section, the integration of multiple markers into a single signature has the potential to improve predictive value over that of a single one. So our study provides a predictive model mainly composed of BMI, D-dimer, neutrophils, and CitH3, which can predict patient DVT and deserves to be explored in future clinical practice.

## Data Availability

The raw data supporting the conclusion of this article will be made available by the authors, without undue reservation.
